# Current Landscape of Non-Small Cell Lung Cancer: Epidemiology, Histological Classification, Targeted Therapies, and Immunotherapy

**DOI:** 10.3390/cancers13184705

**Published:** 2021-09-20

**Authors:** Olga Rodak, Manuel David Peris-Díaz, Mateusz Olbromski, Marzenna Podhorska-Okołów, Piotr Dzięgiel

**Affiliations:** 1 Department of Histology and Embryology, Department of Human Morphology and Embryology, Wroclaw Medical University, 50-368 Wroclaw, Poland; mateusz.olbromski@umed.wroc.pl (M.O.); piotr.dziegiel@umed.wroc.pl (P.D.); 2 Department of Chemical Biology, Faculty of Biotechnology, University of Wroclaw, F. Joliot-Curie 14a, 50-383 Wroclaw, Poland; manuel.perisdiaz@uwr.edu.pl; 3 Department of Ultrastructural Research, Department of Human Morphology and Embryology, Wroclaw Medical University, 50-368 Wroclaw, Poland; marzenna.podhorska-okolow@umed.wroc.pl; 4 Department of Physiotherapy, University School of Physical Education, 51-612 Wroclaw, Poland

**Keywords:** lung cancer, non-small cell lung cancer, epidemiology, histopathology, cancer biology, targeted therapy, immunotherapy, predictive biomarkers

## Abstract

**Simple Summary:**

The abundance and the dynamic of the studies on NSCLC require frequent summaries of the current achievements in the field. In our review, we aimed to update the status of knowledge about NSCLC, combining its epidemiology, classification novelties, tumor molecular basis, and two of the most promising approaches in cancer treatment: targeted therapy and immunotherapy.

**Abstract:**

Non-small cell lung cancer (NSCLC) is a subtype of the most frequently diagnosed cancer in the world. Its epidemiology depends not only on tobacco exposition but also air quality. While the global trends in NSCLC incidence have started to decline, we can observe region-dependent differences related to the education and the economic level of the patients. Due to an increasing understanding of NSCLC biology, new diagnostic and therapeutic strategies have been developed, such as the reorganization of histopathological classification or tumor genotyping. Precision medicine is focused on the recognition of a genetic mutation in lung cancer cells called “driver mutation” to provide a variety of specific inhibitors of improperly functioning proteins. A rapidly growing group of approved drugs for targeted therapy in NSCLC currently allows the following mutated proteins to be treated: EGFR family (ERBB-1, ERBB-2), ALK, ROS1, MET, RET, NTRK, and RAF. Nevertheless, one of the most frequent NSCLC molecular sub-types remains without successful treatment: the K-Ras protein. In this review, we discuss the current NSCLC landscape treatment focusing on targeted therapy and immunotherapy, including first- and second-line monotherapies, immune checkpoint inhibitors with chemotherapy treatment, and approved predictive biomarkers.

## 1. Introduction

**Figure 1:**
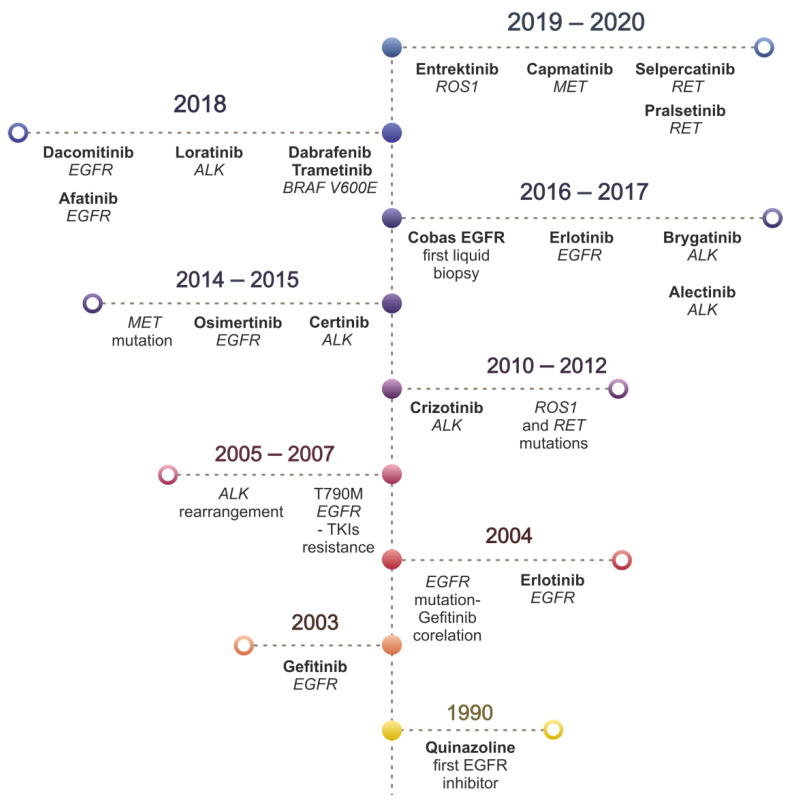
Development of targeted therapy in NSCLC. Over the last decade, there has been an acceleration in the emergence of new inhibitors approved in NSCLC targeted therapy. The approval dates of the inhibitors in the treatment of NSCLC refer to the approvals issued by the Food and Drug Administration (FDA), Silver Spring, MA, USA.

The status of non-small cell lung cancer (NSCLC) is a dynamically evolving landscape. Over the past decades, the advancement of knowledge, the discovery of new drugs, and the diagnostic possibilities have grown exponentially, setting new standards in oncology (Figure 1). Such improvement resulting from continuous technological development allows us to get insights into the molecular mechanisms of cancer cells. NSCLC, targeted by hundreds of research groups, is the infamous winner of the world’s epidemiological statistics on cancer [1,2]. The growing number of patients over the last decade has demanded putting more effort into cancer research, which has resulted in a better understanding of the biology of lung cancer. This knowledge allows us today to practice personalized medicine in which the therapeutic decision depends on the characteristics of the cancer of individual patients.

A breakthrough discovery in lung cancer pathogenesis solidly strengthened the trend of personalized medicine in the treatment algorithms. In 2004, almost simultaneously, two research groups published results confirming the correlation between the effectiveness of a drug (gefitinib) inhibiting the activity of a mutant receptor protein and the presence of mutations in the gene encoding this receptor in treated patients [3,4]. The increased knowledge on the epidemiology and the biology of key mutations has a significant impact on the effectiveness of selected therapy [5]. An important revolution in the clinical approach is the possibility of sequencing the genome of neoplastic cells as a permanent diagnostic element in patients with lung cancer [6,7,8,9,10,11,12,13]. Therefore, due to the achievements of genetics, the existing histopathological classification of lung cancer has been thoroughly rebuilt, adjusting the diagnosis to the current knowledge. Thanks to the introduced changes, a diagnosis of the histopathological subtype is a crucial step in diagnostic algorithms and selecting the treatment regimen [7,14,15]. Although the modifications changed the classification of lung cancers, the definition of NSCLC maintains its clinical significance. The diagnosis of NSCLC and the recognition of its subtype are also prognostic and predictive factors. Both indicators are of great importance for patients since only 2%–20% of patients with NSCLC survive five years from diagnosis [2,16,17]. The main issue is the tumor heterogeneity often observed. This phenomenon refers to the multitude of genetic variants occurring within a single tumor and their variance over time [18,19]. Constant differentiation of new molecular subpopulations complicates diagnostics and the choice of the appropriate treatment. Moreover, the list of known lung cancer genotypes continues to be fueled by newly discovered variants. Thus, the dynamic of lung cancer is one of the biggest challenges in modern biology and medicine [20]. A promising therapy emerged since 2010 when the clinical benefit of immunotherapy was demonstrated [21].

This review summarizes the recent advances in NSCLC and details our focus on epidemiology, the latest histopathological classification, lung cancer heterogeneity, targeted therapy, and immunotherapy. The treatment perspectives in targeted therapy of the best-known genotypes of NSCLC, the approved immunotherapies, and the predictive biomarkers are also reviewed.

## 2. Trends in Epidemiology

The most recent global report on the epidemiology of neoplastic disease states that lung cancer has the highest mortality among 36 cancer types considered, and it is the second most frequently diagnosed cancer type in the world [1,2]. In 2020, based on data from 185 countries, the approximate number of diagnosed cases was estimated at 2,206,771 (11.4% of all cancers), while mortality was 1,796,144 (18.0%) [2]. The mortality is associated with a high degree of malignancy and late diagnosis. As many as 65.33% of men diagnosed with lung cancer are in the advanced local stage (stage III) or present metastases (stage IV) [22,23]. Unfortunately, we still observe a lack of reliable markers for the early stage of the disease [24,25]. However, recent research focused on miRNAs (microRNAs), which have a potential diagnostic value. Their detection in combination with tomography shows a significant increase in the effectiveness of the diagnosis [26]. For example, a phase I/II biomarker study identified two potential miRNAs (miR-15b and miR-27b) that differentiated NSCLC patients from healthy controls with a specificity of 84%, a sensitivity of 100%, a negative predictive value of 100%, and a positive predictive value of 82% [27]. A meta-analysis showed that miR-210 and miR-21 could be used as a diagnostics tool for NSCLC [28]. A recent study generated 2588 miRNAs profiles from a large sample set. The authors identified the miR-17-3p as the best single miRNA for detecting lung cancer with a cross-validation score of 0.9087. Furthermore, the combination of miR-1268b and miR-6075 achieved the best accuracy in the discovery set (cross-validation score of 0.9904) [29].

Moreover, the age of the diagnosed patients is noteworthy. Reports consistently indicate that the incidence of lung cancer over 45 years of age increases dramatically [1,2,5,30]. Hence, specific and effective screening tests are of utmost importance for people over 50 years old.

Although trends in the global incidence continue to rise, the number of new lung cancer cases has been observed to increase or decrease depending on the region. It happens especially among men in highly developed countries such as the United Kingdom, the United States, Australia, New Zealand, Singapore, Germany, the Netherlands, Uruguay, and the Scandinavian countries. Additionally, in developing countries of Eastern Europe, a slow decline in the disease has been also observed [1,2]. During the 1980s in the United States, the lung cancer incidence level reached a plateau for men and has steadily declined [31]. However, the growing number of cases in women is alarming [22,32]. The trends in cigarette smoking shape the patterns of incidence rates observed in particular populations over the decades. In Central Europe, there is a significant gender discrepancy in the values of risk factors for developing lung cancer and mortality. The risk rates are, respectively, 6.29 and 5.67 in men, while in women, they are 1.52 and 1.20, respectively [1]. In men, the risk of disease or death is one of the highest in the world, reflecting the sensitivity to exposure to carcinogenic substances (including tobacco) in the population of Central European men over decades of the last century. Although cigarette smoking remains the leading cause of lung cancer, statistics shows that 12% of people diagnosed with lung cancer have never smoked cigarettes [33]. Interestingly, higher frequency of EGFR mutations was genotyped in never smokers (42.5%) compared to current (4.9%) or former smokers (13.5%) [34,35]. Comparing the incidence of lung cancer in Chinese and French, the rates are 22.8 and 22.5 per 100,000 women, respectively. Although the incidence is at the same level, it is worth noting that the percentage of smokers is much lower among Chinese women than among French women [1]. In the case of China, high exposure to smoke from coal combustion is considered a factor. Thus, air pollution is a significant predisposition to lung cancer [5,36,37].

**Figure 2:**
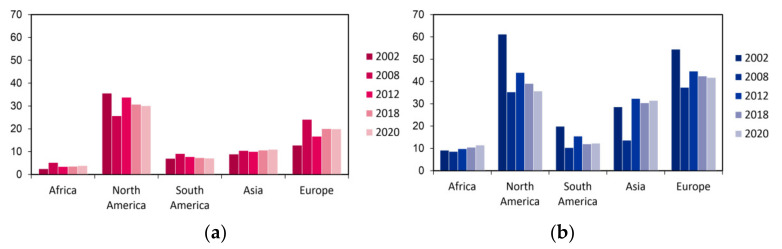
The number of people diagnosed with lung cancer per 100,000 inhabitants. (**a**) Incidence among women concerning world regions in 2002–2020; (**b**) Incidence among men concerning world regions in 2002–2020 [1,2,39,40,41].

Important elements shaping the epidemiology of lung cancer are geographic and economic factors (Figure 2). The cumulative risk of death from lung cancer is not unequivocally followed by mortality value. In North America and Western Europe, the cumulative risks amount to 4.27 and 4.25, respectively, while in the rest of Europe, risk is estimated to be lower, ranging from 3.54 to 3.67 [2]. The advancement in the medical care undoubtedly affects the effectiveness of cancer treatment [38]. Hence, in North America, despite the highest risk of disease, the mortality rate is lower than in Western Europe or East Asia (2.64, 3.04, and 3.36, respectively) [2].

To conclude, despite the local decreasing trends in morbidity and mortality, the incidence rate of lung cancer is continuously increasing worldwide (Figure 2). Therefore, there is a growing need for new solutions, starting from anti-smoking education, counteracting environmental pollution, and ending with the development of innovative diagnostic and therapeutic methods.

## 3. Advancements in Histopathological Classification

In 2015, the World Health Organization (WHO) published a new histological classification of lung cancer, which is a direct result of the achievements of molecular biology that are modeling the current clinical procedure [14]. Three main histological types were maintained: adenocarcinoma (AD), squamous cell carcinoma (SqCC), and neuroendocrine tumors. The other types distinguished were: large cell carcinoma (LCC), adenosquamous carcinoma, sarcomatoid carcinoma, and other unclassified cancers. In addition, the classification of proliferative changes in the lungs includes types of rare occurrence: salivary gland-type tumors, papillomas, adenomas, mesenchymal tumors, lymphohistiocytic tumors, tumors of ectopic origin, and metastases to the lung [42] (Table 1). The most important changes in the classification include (i) reorganization of the group of adenocarcinomas; (ii) restriction of features that classify lesions as large cell carcinoma; (iii) the distinction of a group of neuroendocrine hyperplasia; (iv) change of nomenclature of variants of squamous cell carcinoma [15].

The group of adenocarcinomas was systematized depending on the invasiveness of the lesions. They start from pre-invasive (including adenocarcinoma in situ) through minimally invasive and end with invasive lesions. Among the latter, many variants existed in the 2004 classification. Furthermore, the issue of poorly differentiated neoplastic lesions often diagnosed as large-cell carcinomas was also resolved. Currently, tumors showing a positive immunohistochemical reaction with pneumocyte markers, i.e., the thyroid transcription factor-1 (TT1) or napsin, are no longer classified as LCC. The presence of at least five spots with increased mucus production (the presence of mucin granules in the cytoplasm of more than five cells in the field of view) classifies the lesion into the group of adenocarcinomas. In the absence of such observations, the diagnosis is a squamous cell carcinoma. The modification resulted in a significant decrease in the number of diagnoses of large cell carcinoma, which went down to 1% of the total number of cases [19,43]. Another issue that was taken into account was the classification of tumors with epithelial and non-epithelial origin characterized by neuroendocrine function. Previously dispersed among many subtypes, now, they form a common histological subgroup: neuroendocrine tumors. Interestingly, there is small cell lung cancer (SCLC) included as a subtype of neuroendocrine tumors, which previously functioned independently.

To clarify difficulties in the nomenclature and increase the usefulness of the classification, changes in the systematics of squamous cell carcinomas were introduced. Currently, we distinguish keratinizing, non-keratinizing, basal cell carcinomas, and pre-invasive lesions (squamous cell carcinomas in situ). The variant of small cell squamous cell carcinoma that was easily confused with small cell carcinoma has been abandoned. Moreover, the algorithm for classifying tumors as squamous cell carcinomas has been simplified. The condition for this to happen is the detection of squamous markers, i.e., p40, p63, or cytokeratin 5/6 [44]. These markers qualify the lesions as squamous cell carcinomas also in the absence of keratinization, which allows distinguishing them from adenocarcinomas that present a morphology similar to squamous cell carcinomas [15,42].

The scientific community positively received the updated classification [15,45]. However, pathologists indicate difficulties in the differential diagnosis of adenocarcinomas in situ and those with minimal invasion. It was shown that the assessment based on standard morphology is relatively subjective, emphasizing the need to refine the definition and introduce additional assessment markers [46].

The current diagnostic recommendations emphasize the value of immunohistochemical evaluation (IHC) [15,42]. The vast majority of diagnoses are based on a small amount of tissue, since only a biopsy can be performed in patients in advanced cancer stages [47]. IHC has become the basis for differential diagnosis. In the case of unclear morphology of the cellular component or its heterogeneity, IHC allows identifying the histological type of a lesion. It seemed that the histopathological classification of lung neoplasms had only prognostic significance. Currently, the value of histological assessment has increased significantly, becoming an indispensable element of diagnostic procedure algorithms. Strong emphasis on the molecular nature of the neoplasm dictates the necessity to perform costly tests to detect mutations. Thus, accurate histological diagnosis has become crucial, since each histological type of NSCLC is associated with a characteristic range of gene mutations [12,48,49]. IHC is facing a challenge that targets new biomarkers with higher precision and specificity in the diagnosis of key histological types.

## 4. Genetic Basis of NSCLC Heterogeneity

**Figure 3:**
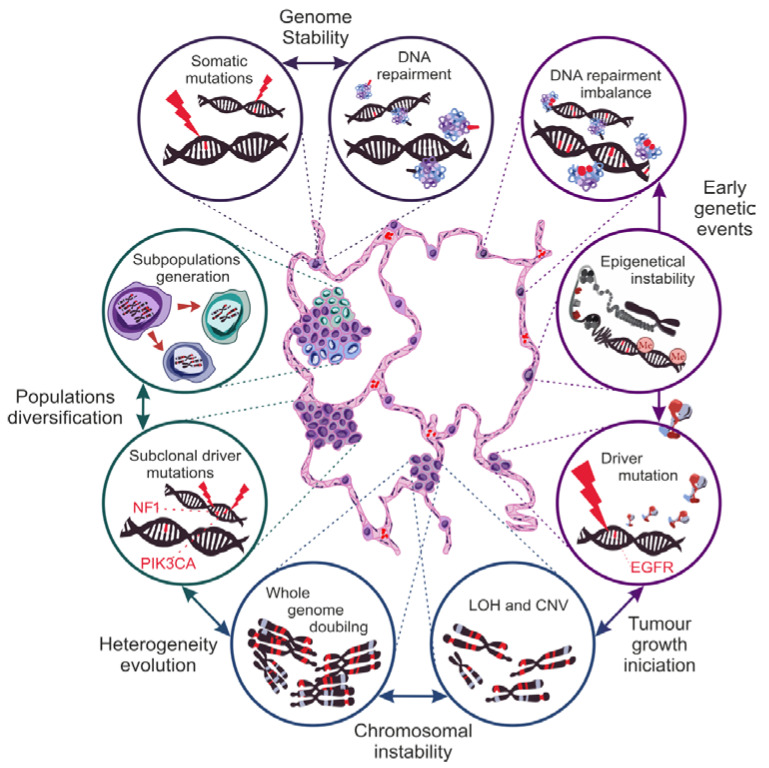
Genetic basis of neoplastic transformation of lung cells and heterogeneity of NSCLC (non-small cell lung cancer). In normal lung tissue, the cell’s genome is kept in balance between mutation occurrence and repair. When endogenous or exogenous factors disrupt this balance, genetic instability occurs, which initiates the pre-initiation phase. During this time, the increasing instability of epigenetic control and the occurrence of new mutations change the activity of the molecular mechanisms. For neoplastic transformation of the cell, occurring changes need to accumulate and cause defects at the chromosome level (initiation phase). Afterward, the whole genome doubling of clonal cells leads to the development of separate populations with different genotypes. Carcinogenesis enters the progression phase, which results from the formation of cells with increased proliferation and invasiveness, triggering the metastasis formation.

The heterogeneous nature of the composition and the growth of NSCLCs is the main obstacle in the therapy of patients in the advanced stage of the disease. All stages of carcinogenesis, from pre-initiation to progression, must be examined to elucidate the underlying causes of this phenomenon. Unfortunately, the multi-layered nature of NSCLC evolution is like a Gordian knot, remaining unsolved. Nevertheless, our knowledge of NSCLC biology is growing, leading to improved conclusions. The first is that genetic disorders are the basis of the neoplastic process [50,51]. Although carcinogenesis begins with a genetic mutation, it should be remembered that a single mutation is not enough for a neoplastic transformation (Figure 3). A pivotal factor in carcinogenesis is increasing genetic instability [52,53,54,55]. By genetic instability, we understand the variability in the severity of disturbances in DNA structure between generations of cells in a given population. Genome instability is, in a sense, a natural phenomenon inherent to the aging process of cells, which, in healthy tissues, manifests itself as somatic mosaicism [56]. On the other hand, tumorigenesis exacerbates it, generating variability at chromosome levels, epigenetic processes, or microsatellite structures.

The stabilization of genetic processes depends on the balance between the intensification of genetic mutations and the cell repair mechanism. In carcinogenesis, the significance of the disturbances in the DNA mismatch repair (MMR) during replication was proven. Tissue analysis of 77 primary NSCLCs showed that more than three-fourths of neoplastic lesions display impairment of the expression of the proteins responsible for MMR repair mechanisms, i.e., bMLH1, hMLH1, and hMSH2, confirming their participation in the pathogenesis of NSCLC [57].

The microsatellite instability (MSI) measurement is used to determine the level of genome instability. Microsatellites are cyclic repeats of several base pairs in the non-coding regions of DNA. By detecting specific MSI phenotypes (depending on the intensity of variance in length and the mutation of microsatellite sections), we can assess the level of dysregulation of the DNA repair processes and, indirectly, the predisposition of cells to neoplastic activation [55]. Several areas of chromatin were located as markers of MSI severity specific to lung tissue cells (on chromosomes 2, 5, 8, 10, 11, and 17). MSI was identified in 68% of NSCLCs that correlates with the stage of disease advancement and the survival time. Moreover, MSI is a strong probability indicator of the recurrence or the initiation of new tumors growth [58,59]. In addition, analysis of MSI in neoplastic tissue allows identifying subclonal populations indicative of the level of tumor heterogeneity. The variation in the intensity of DNA methylation or the occurrence of dysfunctional histone modifications are called epigenetic instability. The global hypomethylation of DNA and the local hypermethylation of the promoters of specific genes result in the disturbance of the proper course of signaling pathways, modulating the intensity of their activity. In contrast, the importance of the acetylation of histone proteins lies in the disorganization of the chromatin packaging system. The exposure of certain regions encoding specific genes leads to a change in the intensity of their expression, which, over time, induces a higher frequency of mutations [54,60]. The epigenetic modification profiles occurring in neoplastic cells are strongly correlated with their histological lineage, creating chromatin mutation patterns characteristic of specific NSCLC subtypes [61]. In addition, the impairment of the APOBEC family enzymes that convert cytosine to uracil during RNA transcript editing leads to an increased mutation accumulation. The assessment of the areas of neoplastic transformation within the lungs and the associated lymph nodes reveals the presence of point mutations characteristic of the APOBEC enzyme [62,63]. Based on the analysis of the genomes of thousands of tumors, including NSCLCs, a specific mutation pattern was established, which is a kind of signature of the defect of the APOBEC protein. This finding confirms APOBEC participation in the development of neoplastic changes in the lungs [19,63]. Neoplastic patterns of genome distortion are also observed in tumor margins, showing normal morphology [64]. Cancerization in the area of the tumor margin tissue is called “field cancerization”. The changes relate to epigenetic modifications and, consequently, the weakening or the intensification of protein expression while maintaining the correct morphology of cells. The observation of lung tissue in people subjected to long-term exposure to carcinogens showed the presence of numerous “field defect” foci in the bronchial tree [65]. Therefore, recurrences often observed in lung cancer or multifocal primary lesions result from the presence of multiple areas of “field defect” and their molecular nature [26,66,67]. The development of epigenetic instability associated with a specific pattern of protein expression dysregulation may be (but not always) a direct trigger of the neoplastic transformation process.

However, it determines its direction depending on the histological type, constituting a solid basis for the initiation of oncogenic activation [60]. The molecular basis for the evolution of neoplastic processes is best known in adenocarcinomas of the lung. Thus far, specific genetic changes responsible for the initiation, the promotion, and the disease progression have been found. The presence of the mutant EGFR (epidermal growth factor receptor) and the K-Ras (Kirsten rat sarcoma viral oncogene homolog) proteins is often observed in the majority of cells of subsequent generations of clones. Hence, they qualify as driver mutations, responsible for the initiation of neoplastic growth, which makes them important as therapy targets [68]. However, it is worth paying attention to the possibility of the simultaneous development of several primary tumors with a different driver mutation basis, which complicates the choice of targeted therapy. In such patients, later metastases originated most often from one primary tumor, precisely from one of its clonal subpopulations [69]. This confirms the importance of monitoring the molecular evolution of NSCLC over time. A prospective study of 100 NSCLC patients in the TRACERx project used the discovery of the presence of circulating tumor DNA (ctDNA) in the blood to trace genetic variation over time. It was shown that driver mutations may develop during the isolation of a subpopulation of cells, initiating the emergence of a cell line with a new genotype, or they may exist only as a “passenger mutation”, not acting as a driver. Mutations of NF1, PK3CA, and KRAS genes are common driver mutations. Nevertheless, their occurrence is not observed in the whole tumor but at the level of specific cell subpopulations, suggesting their appearance at the stage of tumor growth promotion [19].

Key mutations cannot initiate the neoplastic transformation on their own. The development of a clonal cell population also depends on the level of chromosomal instability (CIN), defined as the variance of the number of chromosomal structure impairments in the cell population of a given tissue [53]. One of the subtypes that drive carcinogenesis is the loss of heterozygosity (LOH). The phenomenon consists of silencing or losing alleles encoding correct proteins, favoring alleles burdened with mutations [59]. For instance, in several lung tumors, the p53 suppressor activity was abolished due to the LOH of *TP53* alleles [70]. LOH together with unbalanced duplications of mutant alleles (copy number variations, CNVs) of genes lead to allelic imbalance (AI). The presence of AI on oncogenic genes gradually modulates the activity of proteins that control cell division, which results in the accumulation of subsequent mutations. Both the driver mutations and the CIN work together in an endless loop. Mutations appear one at a time but are fixed thanks to CIN mechanisms. In the cell, the proportion between normal and mutated protein variants changes until there is a significant predominance of impaired proteins, leading to the activation of the carcinogenesis process [71]. However, the initiation of carcinogenesis does not slow down the processes responsible for genetic instability. A comparative analysis of individual subclonal populations showed the variable occurrence of LOH in genes responsible for chromatin remodeling, histone methylation, and response to DNA damage, which led to the formation of new cells genotypes independent of the driving mutation [19]. This observation confirms that CIN is the driving force behind the initiation and the promotion of heterogeneity in lung tumors [72]. Yet, the decisive event is the phenomenon of “whole-genome doubling” (WGD). Genome duplication is considered to be of great importance in tumors with advanced LOHp; especially, it is observed most often in lung cancer. Moreover, it was shown that the mechanisms of natural selection, counteracting the increasing homozygosity of cells, function only until the genome doubles [73]. In addition, alterations in cancer genes that occurred before and after than WGD were related to tumor initiation and progression, respectively [19]. WGD is a turning point in the formation of new subclonal populations, since strengthening all genetic changes occurring in a single cell allows for the differentiation of a genotypically and a phenotypically new cell population. By overcoming one of the milestones of lung carcinogenesis, tumor growth accelerates. The appearance of polyploid cells, characterized by their invasiveness (enhanced proliferative activity acquired as a result of increasing CIN), leads to the final stage of carcinogenesis: progression.

The evolution of lung cancer is still not fully understood, and only a few publications addressed this complex subject [19,62,74,75,76,77,78]. As a result of cell aging, oxidative stress, exposure to mutagenic factors, or hypoxia, the replication machinery generates a higher number of errors [79,80,81,82,83]. Unrepaired genes mutations, decreased allelic heterozygosity, and quantitative changes in gene expression modulate the activity of the proteins responsible for cell division. When key mutations remain unattended due to a growing deficiency of the repair mechanisms, alternations of the most important signaling pathways are triggered. There is a hypothesis that formation of the neoplastic cell protoplast occurs under the natural selection, where a cell with a specific compilation of genetic aberrations survives despite environmental pressure coming from the processes controlling cell proliferation [54,84,85]. Generations of clones proliferate, and the ineffective mechanism regulating the course of cell division over time results in WGD, this being the first step towards the diversification of subclonal populations. From that point onwards, mutations lose their importance, and CIN takes control of the evolution of heterogeneity.

## 5. Overview of Targeted Therapy for NSCLC

The lack of a uniform pathomechanism of NSCLC results in the lack of a standardized treatment method. The issue arises from the wide range of driver mutations, and, hence, the number of cancer cells genotypes that initiate and maintain the process of carcinogenesis in the lungs.

**Figure 4:**
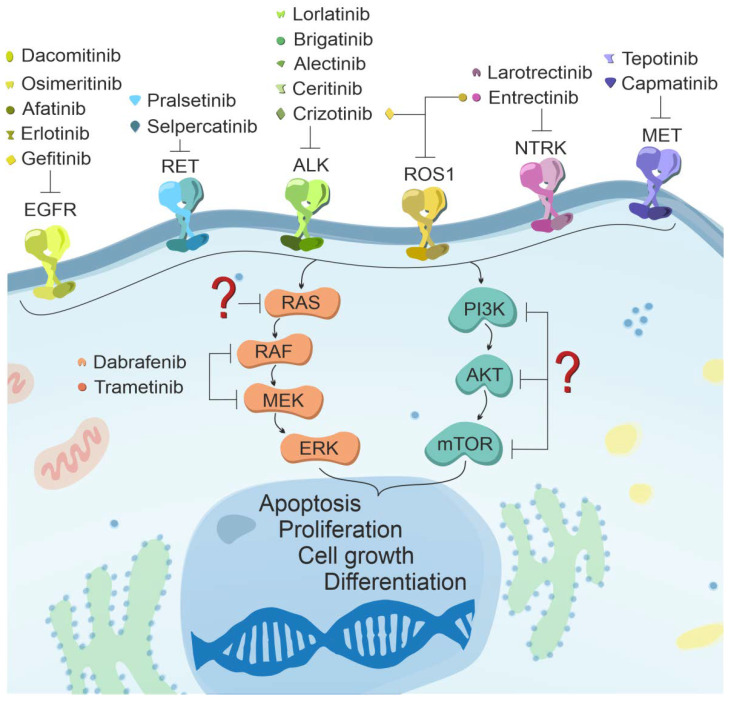
Schematic representation of the leading signaling pathways for which FDA-approved inhibitory substances were developed thus far (EGFR—epidermal growth factor receptor; HER2—human epidermal growth factor receptor 2; ALK—acute lymphoma kinase; ROS1—c-ros1 oncogene; NTRK1—neurotrophic tyrosine kinase receptor type 1; MET—tyrosine-protein kinase Met; RAS—rat sarcoma 2 viral oncogene homologs family; RAF—proto-oncogene c-RAF; MEK—mitogen-activated protein kinase; ERK—extracellular signal-regulated kinases; Pi3K—phosphoinositide 3-kinases; AKT—protein kinase B; mTOR—mechanistic target of rapamycin kinase).

Carcinogenesis is initiated when an irreversible and heritable mutation occurs in one of the key proteins that control any vital cell functions (proliferation, adhesion, DNA repair, etc.). However, later on, the promotion of neoplastic change depends on the development of oncogenic patterns of gene expression (oncogene addiction) in subsequent generations of cells. The goal of therapy is to be able to disable them effectively. One strategy called targeted therapy aims to inhibit the activity of key proteins resulting from driver mutations (Figure 4). Nevertheless, despite the growing amount of research aimed at better understanding the cancer process and finding effective inhibitors of target proteins, our capabilities remain insufficient to treat each patient effectively. One main goal of this review was to update and summarize the knowledge of available targeted therapies for different NSCLC genotypes. Current and developing treatments for selected driver mutations are discussed as well as milestones in the progress of effective targeted therapy.

### 5.1. Protein Genes from the Epidermal Growth Factor Receptor (EGFR) Family

The *ERBB1* gene mutation was the first discovered mutation in NSCLC. Nowadays, it is the most common target of targeted therapy, since as many as 20% of patients with lung adenocarcinoma carry it [34]. The gene codes for the EGFR protein (ERbB1, HER1), which is a membrane receptor from the group of tyrosine kinases. The binding of the ligand to the extracellular domain of the receptor becomes possible through the formation of homo- or heterodimers (with other proteins from the ERBB family, i.e., HER2, HER3, or the MET protein) [86,87]. This results in intracellular signal transduction and the auto-phosphorylation of tyrosine residues, which activate EGFR-dependent signaling pathways responsible for the control of the cell cycle [88]. In neoplastic cells, impaired EGFR function most often results from *ERBB1* gene overexpression, increased gene copy number, or the presence of a mutation [89]. Mutated proteins do not degrade and form dimers with high affinity, which leads to the unlimited activity and the autonomy of the receptor [86]. It triggers inhibition of apoptotic pathways, continuous proliferation, and blockade of the gene expression patterns that define cell differentiation [3,4,88]. Moreover, it contributes to the initiation of angio- and lymphangiogenesis processes in the neoplastic tumor, which is the first step towards the invasion of cancer cells and the formation of metastases [90,91]. The identification of a mutation of EGFR-encoding genes in patients, even with advanced NSCLC, is considered a favorable prognostic factor. The presence of mutated EGFR most probably means that the tumor is sensitive to tyrosine kinase inhibitors (TKIs) [92]. Unfortunately, tumor recurrence or TKI resistance usually appear. As a result, up to now, three generations of TKIs were developed. The T790M mutation in the *ERBB1* gene is responsible for TKI resistance. The mutant blocks the specific binding of first and second-generation TKIs to EGFR [93]. Two major explanations of the development of resistance were described. In one, the mutation has not been diagnosed, e.g., due to its presence in a small subpopulation of cell clones. The second results from the appearance of the mutation during treatment de novo. Osimertinib is the only third-generation TKI approved by the Food and Drug Administration (FDA) that irreversibly blocks the receptor burdened with the T790M mutation as well as most common EGFR mutations. Due to its ability to cross the blood–brain barrier, osimertinib received accelerated approval in 2015 for patients with NSCLC metastases with the T790M mutation [94]. Shortly after, the results of the AURA3 clinical trials achieved full approval of the drug, showing an overall response rate of 72% and a 10.1 month progression-free period versus 31% response and 4.4 months progression-free in patients treated with chemotherapy [95].

Moreover, the decision to introduce liquid biopsies (blood tests or tests for free circulating nucleic acids from cancer cells) for the presence of the T790M mutation—cobas^®®^ EGFR Mutation Test v2 [95]—largely contributed to the success of these studies. Although the test accelerated the possibility of a therapeutic decision-making, later studies showed that the negative results may be false in up to 30% of the cases. [96,97]. Hence, the lack of detection of the T790M mutation in the blood requires confirmation by analyzing the tissue material taken directly from the tumor. However, nowadays, less attention is paid to identify such mutations, since osimertinib is used as a first-line therapy [98]. Its superiority was confirmed by the latest results of the FLAURA clinical trials where osimertinib was compared with first-generation TKIs. The results showed increased progression-free and overall survival (OS) when using osimertinib as a first-line therapy. The medians were 38.6 and 31.8 months for third- and first-generation drug (gefitinib or erlotinib) groups, respectively [99,100]. This led to the approval of osimertinib for first-line treatment in 2018. Moreover, the efficacy of osimertinib was tested against less common mutant EGFR variants, also obtaining satisfactory results [101]. However, there are also significant disadvantages in using third-generation TKIs. One of them is the occurrence of severe toxicity (irAE—immune-related adverse events) during osimertinib therapy. In this sense, toxicity was found in 15% of patients who had previously undergone immunotherapy [102]. With at least a year break between the above-mentioned therapies, the side effects were reduced, which proves the very long half-life of the checkpoint inhibitors used in immunotherapy [103]. More importantly, the simultaneous use of osimertinib and durvalumab (immunotherapy drug) increased the percentage of patients who developed interstitial lung disease to 38%. Moreover, the response rate to treatment with the combination of drugs was lower than that of osimertinib alone, i.e., 64% vs. 80% [104].

Despite significant advances in the knowledge of the *ERBB1* gene mutations, new questions continue to arise, especially around the process of acquiring resistance. It is worth mentioning that recent reports indicated cancer transition into neuroendocrine subtype (from 3% to 10% of adenocarcinomas), the treatment of which is less effective [105,106,107]. Furthermore, this differentiation may lead to the development of resistance to third-generation TKIs [108]. TKI resistance is the “Achilles heel” of targeted therapy. However, it should be remembered that a better understanding of the molecular basis of the *ERBB1* gene mutation led to significant success, increasing the overall survival of patients with the above-mentioned mutation, which accounts for approximately 20%–23% of patients with lung adenocarcinoma [109].

The *ERBB2* (HER2—human epidermal growth factor receptor 2) gene mutation encoding a receptor from the EGFR family occurs in 3% of lung adenocarcinomas [110]. Thus far, clinical trials focusing on the use of monoclonal antibodies showed a 44% response rate, achieving a maximum reduction of 69% in the mass of neoplastic lesions [111]. On the other hand, in other studies, a lack of efficacy of these antibodies in combination therapy with chemotherapy in patients with *ERBB2* gene amplification was found [112]. Hence, clinical trials are currently using inhibitors against the most common mutation of HER2. In vitro studies carried out on organoids showed the anti-tumor activity of pyrotinib resulting from the inhibition of the tumor cells growth. Therefore, pyrotinib was qualified for clinical trials, where it showed a response rate of 53.3% in a group of 15 patients [113]. On the other hand, preliminary phase I results on an inhibitor TAK-788 did not show satisfactory results. There were only 3 out of 14 patients that showed a partial response to treatment. The last compound with therapeutic potential for *ERBB2* mutation is poziotinib, which achieved an overall response rate of 42% in the second phase of clinical trials [114].

### 5.2. Anaplastic Lymphoma Kinase (ALK) Receptor Gene

The physiological function of ALK is not yet fully understood. While many studies assessed individual components of the downstream ALK signaling pathways, their selectivity does not allow for a comprehensive understanding of the role of the ALK receptor. Although the exact molecular mechanisms are unknown, the existing knowledge shows a set of cellular processes in which the ALK receptor is involved (control of cell cycle, cell growth, cell differentiation, and anti-apoptotic signaling pathways) [115]. Thus far, three main activation mechanisms of the oncogenic receptor have been identified. These are gene amplification, fusion with a gene of another protein, or mutation in the sequence of the *ALK* gene itself. The location for *ALK* is considered very common for chromosomal translocation, leading to fusion with another gene and, thus, the production of linked proteins with altered properties. This mechanism of *ALK* genetic aberration is most often observed in lung adenocarcinomas, and its incidence is approximately 5%–6% [116,117]. Until now, among the 22 fusion partners, the most frequently identified concerned the EML4 protein (echinoderm microtubule-associated protein-like 4), which leads to the development of adenocarcinoma [118]. Other variants observed in NSCLC are fusions with the following proteins: KIF5B (kinesin family member 5B), TFG (TRK-fused gene), KLC1 (kinesin light chain 1), PTPN3 (protein tyrosine phosphatase non-receptor type 3), and STRN (striatin). The oncogenic activity of the ALK protein results from the acquired autonomy during fusion [115]. A conformational structural change results in permanent phosphorylation and activation of the kinase domain, bypassing the ligand binding-induced ALK protein dimerization step [119]. In 2013, seven years after the discovery of the EML4-ALK fusion as a driver mutation in lung adenocarcinomas, the efficacy of the first targeted TKI, crizotinib, over chemotherapy was documented [120]. Subsequently, due to the emergence of acquired resistance, two therapeutics obtained accelerated approval for second-line treatment: ceritinib and alectinib, both second-generation TKIs. Finally, based on the promising results of the clinical trials ASCEND-4 and ALEX, they were introduced as first-line drugs [121,122,123,124]. Another second-generation TKI with proven therapeutic potential for patients who developed resistance to crizotinib was brigatinib. Clinical trials using brigatinib exhibited a 53.6% overall response rate and a 73.3% response rate to the treatment of active measurable brain metastases. These results permitted the drug to be approved for the treatment of patients with disease progression [15]. Moreover, a study was performed in which patients who had not previously received TKIs crizotinib and brigatinib were compared. The new generation inhibitor showed a slightly higher overall response rate. Nevertheless, brigatinib showed 76% versus 26% response rate to treatment with crizotinib regarding intracranial metastases. Thus, obtained results proved a significant intracranial penetration and therefore promoted brigatinib as an effective first-line drug in advanced stage cancer [125]. Unfortunately, there are still no studies comparing brigatinib with other second-generation TKIs and, thus, no conclusion can be derived. Notwithstanding, the FDA approved brigatinib for first-line treatment in 2020.

The presence of second-generation TKIs as first-line drugs did not avoid acquired resistance to targeted therapy, attributed to point mutations in the *ALK* gene. It is noteworthy that some mutations are TKI-specific, and the same mutations can appear in response to different TKIs. One example is the G1202R mutation, which can be triggered by any TKI generation [126]. As the G1202R mutation is resistant to all TKIs of the first and the second generations, the FDA approved a third generation TKI to use in the second and the third lines of treatment: lorlatinib. Lorlatinib is a macrocyclic compound with a broad spectrum of inhibition of mutant variants. A clinical trial showed a high response rate (90%) in treatment-naive patients with *ALK* gene fusion [127]. In patients who had failed second generation TKI treatment, lorlatinib achieved a 69% response rate. Although the results obtained are very promising, common side effects of lorlatinib were observed. More than 80% of patients showed hypercholesterolemia, 60% of them showed hypertriglyceridemia, and 3% had to discontinue treatment due to other severe side effects [128]. Meanwhile, the availability of a wide range of TKIs for patients with *ALK* gene fusion seems to be very beneficial; the constant genetic evolution of NSCLC requires a personalized approach. A perfect example is the clinical case of a patient with the presence of the ELM4-ALK protein fusion with advanced lung cancer and liver metastases. The use of crizotinib (first generation TKI) resulted in an early improvement, but after 9 months, tumor progression and liver failure was observed. The patient did not respond to second generation TKIs and chemotherapy. Genotyping showed that resistance resulted from the C1156Y mutation, which is susceptible to lorlatinib. Surprisingly, after the treatment, the patient was re-diagnosed with acquired resistance to lorlatinib. The detection of the coexisting L1198F mutation paradoxically helped the patient, as this mutation increases the binding affinity of the first generation TKI. Re-therapy with crizotinib resulted in rapid regression of metastases and recovery of the liver function, confirmed by computed tomography 6 months after the start of treatment [129]. The success of the discussed case was conditioned by a flexible approach based on the documented properties of new therapeutic substances and the systematic use of molecular diagnostic techniques.

### 5.3. The C-Ros Oncogene 1 of the Receptor Tyrosine Kinase (ROS1) Gene

The ROS1 membrane receptor is an enzyme with a highly homologous structure to the ALK protein and thus follows a similar oncogenic nature (i.e., fusion with another protein). The function of ROS1 proteins is related to the control of cell differentiation and growth. Hence, in NSCLC, when *ROS1* gene rearrangement (most often with CD74, SLC34A2, or FIG proteins) takes place, oncogenic hyperactivity of the protein is observed [130]. Due to the similar structure of ROS1 to the ALK protein, the efficacy of crizotinib in a cellular model was very quickly demonstrated that was subsequently confirmed in clinical trials [131,132,133]. However, the acquisition of resistance was also observed. One of the first described resistance pathomechanisms was a point mutation in the kinase domain G2032R. Interestingly, the mutation was present in all metastases, suggesting that this mutation was an early event in the carcinogenesis before the invasion phase [134]. An alternative resistance mechanism is the cell adaptation to protein activity inhibition that initiates oncogenic patterns of gene expression. Cells become independent of the driver mutation by developing alternative signaling pathways to maintain their proliferation. Resistance to ROS1 inhibition can be mediated by EGFR or RAS activation. For example, in NSCLC cell line HCC78, resistance to ROS1 inhibition leads to cells sensitive to EGFR inhibition [135,136]. In this case, it is recommended to re-genotype the biopsy material for the presence of other protein mutations [137]. Unfortunately, crizotinib also has poor intracranial penetration. Approximately 34%–36% of patients with advanced NSCLC show the presence of brain metastases that is considered to be the most common cause of mortality [138]. Hence, a new drug that inhibits the ROS1, entretinib, was approved in 2019, showing significant activity against intracranial metastases.

Entretinib is an inhibitor of the tropomyosin receptor kinase (TRK), and its mechanism of action is based on the ROS1 receptor dependence on the TRK activity [139]. It is one of the first inhibitors approved under the FDA’s revised policy. Thus, entretinib can be used for specific gene mutations independently of the cancer type. The acceptance of entretinib was based on the results of three independent clinical trials involving 53 patients. Despite the positive results (77% of the overall response rate), it should be mentioned that the inhibitor is also characterized by a high percentage (11%) of severe side effects, including those related to nervous system and cardiovascular disorders. Nevertheless, these effects can be regulated by lowering the drug dose [140]. A choice for patients with intracranial metastases is the previously discussed ALK inhibitor, lorlatinib, which is active against mutations acquired during treatment with crizotinib. In clinical trials, lorlatinib showed a 62% response rate in patients who had not received previous treatment and 35% in those who had received crizotinib. For the remaining ALK inhibitors, only the efficacy of ceritinib was assessed in clinical trials, with an overall response rate of 62% [141]. To conclude, it can be stated that targeted therapy for *ROS1* gene mutation is characterized by relatively low effectiveness. Accordingly, next generations of inhibitors are sought, exemplified by DS-6051b and repotretinib, which showed promising potential in preclinical studies thus far [142,143].

### 5.4. Tyrosine-Protein Kinase MET Gene—MET

The mutation of the *MET* gene, which encodes another protein from the group of receptor tyrosine kinases, occurs in two variants: deletion of exon 14 and gene amplification. Exon 14 aberration reduces the degradability of the protein, which disables the mechanism regulating the number of active MET homodimers. As a result, there is an accumulation of proteins in the cell membrane, and their activity increases. Although crizotinib and cabozantinib are not direct inhibitors of MET, due to the receptor heterodimerization with proteins from the EGFR family, they show a partial inhibition of their oncogenic activity. The retrospective evaluation of 61 cases confirmed 24.6 and 8.1 months overall survival for patients treated with at least one of the inhibitors and non-treated patients, respectively. Such results represent a significant increase in overall survival [144]. In recent years, several MET inhibitors more selectively targeting the mutation have been selected for clinical trials (capmatinib, tepotinib, glesatinib, and savolitinib). Capmatinib received accelerated FDA approval this year. Unfortunately, the variant of the *MET* gene amplification remains without therapy treatment. Its presence was proven to reduce the survival time of patients, which is why it is considered a negative prognostic indicator [144]. Moreover, it is one of the mechanisms of acquired resistance to TKIs in patients with *ERBB1* gene mutation. Studies on a selected small group of 12 people (patients with a medium to a high degree of *MET* gene amplification) showed a 42% response to treatment with crizotinib, while another 42% of patients showed stabilization of the disease [145]. Only one study performed on a small group of 16 patients used an antibody-based inhibitor targeting the *MET* gene amplification (telisotuzumab vedotin). The results of the first phase of the clinical trials showed a low 18.8% response to treatment [146].

### 5.5. Tyrosine-Protein Kinase RET Gene—RET

The RET protein is a membrane tyrosine kinase receptor. RET mutations are most often diagnosed in medullary thyroid cancer. Nevertheless, its mutation leads to the development of NSCLC. Its oncogenic activity is caused by chromosomal rearrangement, which leads to the formation of proteins with altered receptor activity [147]. Its main partner is the KIF5B protein (kinesin family member 5B), which accounts for 62% of all *RET* gene rearrangement variants [148]. To date, there have been approved three RET inhibitors, namely cabozantinib, vandetanib, and alectinib. However, the lack of approval for use in NSCLC meant that they could only be used as a last-line treatment.

Retrospective studies evaluated 165 patients with the *RET* mutation where the overall response rates to cabozantinib, vandetanib, and sunitinib were 37%, 18%, and 22%, respectively. The studies showed a median overall survival of only 6.8 months [149]. It is worth mentioning that RET mutations account for about ~1%–2% of lung adenocarcinomas, which additionally indicates the need to develop new and more effective inhibitors. Recently, two molecules—pralsetinib (BLU-667) and selpercatinib (LOXO-292)—obtained FDA approval for use in advanced metastatic NSCLC. The ARROW clinical trials conducted on 87 patients who had previously received chemotherapy and 27 previously untreated patients showed 56% and 70% response rates to pralsetinib treatment, respectively. On the other hand, the LIBRETTO-001 studies carried out on 105 patients who had previously undergone chemotherapy and 39 previously untreated patients showed 64% and 85% response rates to selpercatinib treatment, respectively [150]. Thus, these new developed drugs have shown promising results.

### 5.6. Neurotrophic Tyrosine Kinase Receptor Type 1—NTRK1

Another protein involved in the neoplastic transformation of lung cells is the tropomyosin receptor kinase (TRK), encoded by the *NTRK1* gene. Under physiological conditions, TRK regulates cell growth and differentiation processes. Their oncogenic activity most often results from fusion with *CD74* or *MPRIP* (myosin phosphatase Rho interacting protein) genes [151]. Although the mutation in the *NTRK1* gene is quite rare (estimated <1%), there are two TKIs targeted to these abnormal receptors. Entretinib and larotrectinib were approved after clinical trials performed on various types of cancer, including NSCLC. Nevertheless, the response rates were 70% and 75% for entretinib (higher than the overall study group—57%) and for larotrectinib (three of whom were complete responders), respectively [152,153]. Unfortunately, there are still no clinical trials comparing the two drugs, and there are no data on the resistance acquisition. Therefore, chemotherapy remains a frequent therapeutic choice for patients with *NTRK1* gene fusion.

### 5.7. V600E Mutation of the BRAF1 Gene (Rapidly Accelerated Fibrosarcoma Homolog B)

The mutation of the B-Raf V600E protein concerns an enzyme that is part of one of the most important signaling pathways: RAS/RAF/MEK/ERK (MAPK/ERK pathway). A meta-analysis of the available data (up to January 2016) on *BRAF1* mutation showed that it occurs in 2.6% of NSCLC patients, while other sources estimated that it accounts for about 8% of lung adenocarcinomas [48,154]. Initially, targeted therapy was based on the use of inhibitors alone (vemurafenib or dabrafenib), but their efficacy was not satisfactory. Their response rates were 42% and 33%, respectively [155,156]. Subsequent studies demonstrated a 63%–64% increase in the response rate when trametinib, a MEK protein inhibitor regulated by B-Raf, was combined with dabrafenib therapy [157]. *BRAF1* mutations are often observed in melanomas, where they are divided into three subclasses due to their different influence on the signaling pathways. The first is the V600E mutation. The second class includes other mutations that result in moderate to high kinase activity, regardless of the activity of the regulatory protein RAS. Finally, the third class includes absent or disturbed kinase activity and other unclassified mutations [158]. Studies on NSCLC cell lines showed that the effectiveness of selected inhibitors of the MAPK/ERK pathway depended on a specific class of *BARF1* mutations, suggesting the introduction of this division into clinical and preclinical studies [159].

### 5.8. KRAS Gene Mutation (Kirsten Rat Sarcoma Viral Oncogene Homolog)

Undoubtedly, the multitude of targeted therapy regimens is a sign of significant advancement in NSCLC treatment. However, the availability of inhibitors with proven efficacy does not correspond to the frequency of given gene mutations in patients. There are no approved inhibitors for patients with confirmed *KRAS* mutation, which accounts for up to 32.7% of NSCLC and 27% of the adenocarcinoma subtype [160,161]. The *KRAS* mutation has been acknowledged as the milestone or the greatest challenge of targeted therapy, and the attempts to develop inhibitors have been compared to a “game of thrones” [162]. Thus, up to now, the inhibitors of KRAS protein remain the most desired small molecules in targeted therapy. Although research is still in the development stage, we would like to review selected therapeutic strategies.

The RAS family is composed of intracellular GTPases, G proteins. They are presented in two forms: active Ras-GTP and inactive Ras-GDP. As an early signal transmitter, RAS controls MAPK/ERK and phosphoinositide 3-kinase (PI3K) signaling cascades, and it is responsible for activating STAT transcription factors, collectively controlling proliferation and apoptosis of the cell [163]. Mutation triggers conformational changes, thus, the enzyme is trapped in its active form, resulting in a permanent transmission of the signal for proliferation. The RAS family is represented by three proteins with a strictly homologous structure: K-Ras (Kirsten rat sarcoma viral oncogene homolog), N-Ras (neuroblastoma rat sarcoma viral oncogene homolog), and H-Ras (Harvey rat sarcoma viral oncogene homolog). Although the discovery of the oncogenic activation of the K-Ras protein in a lung tumor cell line was described as early as 1984 [164], all attempts to find an effective targeted therapy for lung cancer patients were unsuccessful [165,166]. The problem of finding an effective inhibitor is related to the variety of mechanisms present in cancer cells with *KRAS* mutation [166].

First, the detection of the K-Ras mutant is not always related to its dominance (in other words, to its driving character). This means that a mutation can also occur as a co-mutation, a consequence of an oncogenic activation of another gene. However, to achieve an effective treatment, the target should be set up on a driver mutation that causes neoplastic transformation [165]. Moreover, point mutations lead to conformational changes of the protein, changing its activity. The multitude of occurring mutant variants results in a mutation-specific reprogramming of the cancer cell metabolism. This in turn gives us a wide variety of metabolic phenotypes, as seen in cancer cells burdened with the *KRAS* mutations. The discussed heterogeneity of changes in the sequence and, thus, in the spatial structure of the mutant K-Ras explains the lack of possibility to find a universal inhibitor [167]. On the other hand, there is considerable homology between the K-Ras protein and other GTPases associated with tyrosine kinase receptors. The structural similarity relates to the guanine nucleotide binding region: the G-domain. Thus, the lack of appropriate selectivity leads to the function inhibition of other key receptors and the complete disorganization of the signaling pathways also in healthy cells [166]. Another reason for the above-mentioned issue is the occurrence of co-mutations. Mutations in *TP53*, *KEAP1,* and *STR11* genes are mentioned as the most common. The proteins encoded by them influence, among others, the activity of immune system cells in the tumor microenvironment, which impacts the effectiveness of immunotherapy [168]. Moreover, the presence of certain co-mutations defines the metabolic phenotype of a neoplastic cell. An example is the deletion of the *LKB1* gene which, by affecting the expression of the KEAP1 protein, changes the metabolism of the Krebs cycle (TCA). Physiologically, for the proper course of TCA, the availability of glucose is necessary. However, as a result of *LKB1* deletion, neoplastic cells become independent of the process of glycolysis, deriving energy from glutaminolysis [169]. The phenomenon is the basis of an interesting therapeutic strategy [169]. Currently, phase 1 clinical trials have started to check the effect of the glutamine inhibitor telaglenastat (CB-839) in advanced NSCLC [170]. However, the preliminary results of the studies in an animal model showed that the use of CB-839 as monotherapy in lung tumors with the K-Ras mutation did not give satisfactory results; for this reason, it is suggested to include selective inhibitors of glycolysis as well [171].

Another factor contributing to the difficulty of treating *KRAS*-mutated NSCLC is the high AI of the *KRAS* gene. As a result of the deletion of the wild-type allele or the amplification of the mutant variant, the balance between errors in the DNA sequence and the normal genome is lost, which changes the intensity of the transcription and the post-transcriptional modifications. The presence of mutations in most of the alleles is associated with a higher cancer malignancy and a shorter survival time [172].

Point mutations occur most frequently in the *KRAS* gene. This applies to codons 12, 13, and 61, of which the most common mutations are observed in the first ones: G12C (which changes the amino acid sequence in codon 12 from glycine to cysteine), G12V (change from glycine to valine), and G12D (change to aspartic acid), representing 41%, 19%, and 14% of all *KRAS* mutation variants, respectively [161]. Unfortunately, the drugs currently being tested are mainly at the beginning stage (preclinical) of the research. The most promising ones are the selective inhibitors of proteins with the *KRAS^G12C^* mutation. The mechanism of their action is based on the block of the mutant protein in an inactive form. In phase I clinical trials, with the use of AMG 510 and MRTX 849 in patients with the *KRAS^G12C^* mutation, four patients with NSCLC were assessed at the first checkpoint. Both compounds showed the same results. Stable disease was observed in two patients, and there was a partial response in one patient, representing 75% response to treatment [173,174]. At the end of 2020, the first assessment of clinical activity of adagrasib (MRTX 849) in the KRYSTAL-1 study was completed. Presented results demonstrated only 45% partial response. Interestingly, disease control rate was 96% from 51 patients with previously treated *KRAS^G12C^*-mutant NSCLC. Moreover, higher overall response rate—64%—was observed in patients presenting a co-mutation in the *STR11* gene, suggesting the importance of co-mutation diagnostics [175]. In the case of the second compound AMG 510 (sotorasib), the full analysis of data collected during phase II clinical trial was published. Results showed disease control in 80.6% of patients, of which 81% had previously received at least one therapy (chemotherapy or/and immunotherapy). Objective response to the treatment was 37.1%, including 3.3% that achieved complete response. Drug tolerability was moderate, as 69.8% of patients showed treatment-related adverse effects.

Lonafarnib and tipifarnib are specific inhibitors of farnesyltransferase, the enzyme responsible for post-translational modification of the K-Ras mutant. The action of the enzyme is based on the catalysis of binding a hydrophobic farnesyl residue to the K-Ras, anchoring the protein in the cell membrane. Despite the promising results obtained in the preclinical phase, lonafarnib showed a 10% response rate, and disease stabilization was achieved in 38% of patients [176]. The use of tipifarnib also showed a negligible clinical effect despite the strong inhibiting activity of the K-Ras farnesylation. Moreover, this drug showed significant toxicity in treated patients [177]. Nevertheless, in 2018, tipifarnib returned to clinical trials as a treatment for squamous cell lung cancer patients with the H-Ras mutation.

Hence, the complex biology of the RAS mutant variants is not only an obstacle, but it also has great research potential. With the discussed diversity of oncogenic pathways induced by the K-Ras mutants, we observe a multitude of ongoing approaches for therapeutics development. These include (i) inhibition of K-Ras binding to the cell membrane by post-translational modifications, (ii) manipulation in the *KRAS* gene expression processes, (iii) control of protein degradation, (iv) inhibition of GTP binding or binding to effector proteins, (v) attempts to block key metabolic processes, and (vi) use of synergistic inhibitors of proteins related to the K-Ras signaling pathway [178,179]. Although there are many possibilities in the area of treatment of NSCLC caused by mutations in the *KRAS* gene, it seems that an effective and safe therapy is still to be discovered.

### 5.9. FDA Approved TKIs

To date, the FDA has approved seventeen TKIs for NSCLC therapy (Table 2). One of them, crizotinib, can be incorporated in the treatment of two different targets: ALK and ROS-1. Furthermore, TKIs targeting NTRK received approval as tissue-agnostic drugs for cancer therapy.

## 6. Overview of Immunotherapy for NSCLC

Targeted therapies are no longer the only treatment option; immunotherapy (IO) has dramatically modified the NSCLC treatment landscape [180]. The acquired resistance to targeted therapies remains a major and inevitable challenge, and, therefore, new approaches must be considered. Cancer cells have multiple immunosuppressive mechanisms to escape from the immunological response and survive [181]. Therefore, immunotherapy exploits the concept of activating or regulating the immune system to identify and kill cancer cells. To date, one of the main approaches is to develop immune checkpoint inhibitors (ICI) to target pathways used by cancer cells to escape the immune system. Particularly, inhibitors of cytotoxic T lymphocyte antigen-4 (CTLA-4) and programmed death receptor (PD-1) and PD-ligand 1 (PD-L1) checkpoints, which regulate priming and effector phases of T-cell activation, respectively, were approved by the FDA (Table 3) [182]. Clinical trials using other immune checkpoint inhibitors are ongoing, targeting T cell immunoglobulin and mucin-containing protein 3 (TIM-3) (NCT03311412, NCT02817633, NCT03307785) [183,184], lymphocyte activation gene-3 (LAG-3) (NCT03311412, NCT03538028, NCT03156114) [184,185,186], V-domain lg suppressor of T cell activation (VISTA) (NCT02671955, CTRI/2017/12/01 1026) [187,188], human endogenous retrovirus-h long terminal repeat-associating protein 2 (HHLA2), and T cell lg and immunoreceptor tyrosine-based inhibitory motif domain (TIGIT) (NCT04746924, NCT04866017) [189]. Most of the ICIs proved a limited benefit with 10%–20% overall response rates of monotherapy [190]. One of the approaches to improve the ICIs efficiency consists in the development of better predictive biomarkers [191]. Another approach is the combination treatment strategies such as ICIs combinations with chemotherapy [192,193], radiotherapy [194], or TKI [104,195].

### 6.1. Immune Checkpoint Inhibitors

ICIs are compounds that block immunosuppressive mechanisms of cancer cells. There are mainly seven stages, called cancer-immunity cycles (CIC), involving the immune system response to cancer cells [190]: (i) cancer antigens are released from cancer cells, (ii) cancer antigen presentation to T cells, (iii) T cells activation, (iv) T cells trafficking to tumors, (v) T cells infiltration to tumors, (vi) cancer cell recognition by T cells, and (vii) elimination of cancer cells. Cancer cells may evade the autoimmune response by several mechanisms. For example, immune checkpoint molecules such as PD-L1 expressed by tumor cells interact with PD-1 receptors expressed on activated T cells and inhibit T cell activation, promoting tumor immune escape [196].

CTLA-4 was the first known immune checkpoint, expressed on regulatory T cells (Tregs) and on the surface of activated T lymphocytes [21]. During the T cell activation (CIC third step), the receptor protein CTLA-4 competes with CD-28 receptors to bind to the B7-1 and the B7-2 ligands expressed on antigen-presenting cells (APCs) [197]. The higher affinity of CTLA-4 to bind B7 instead of CD-28 inhibits B7-CD-28 binding and suppresses the T cell activation. ICIs of the CTLA-4/CD-28 checkpoint pathway may suppress the CTLA-4-B7 binding, promoting the activation of immune responses [198].

In 2014, the first two ICIs (nivolumab (NCT01721772) and pembrolizumab (NCT01295827)) targeting PD-1 were approved by the FDA for malignant melanoma [199,200], and, in 2015, nivolumab emerged as a novel second-line treatment in advanced squamous cell and NSCLC patients regardless of PD-L1 expression level (CheckMate 017, CheckMate 057) [201,202] (Table 2). Later, other anti-PD-1 antibodies, pembrolizumab and atezolizumab (OAK trial) [203], were introduced as second-line NSCLC therapies, and then pembrolizumab was approved for the first time as a first-line treatment for NSCLC without driver mutations (KEYNOTE-024) [204]. Approximately 25%–30% of NSCLC patients exhibit high PD-L1 expression (tumor proportion score, TPS ≥ 50%) and can benefit from a first-line therapy, for instance, with pembrolizumab [204]. In 2018, durvalumab was FDA-approved as a second-line therapy (PACIFIC) [205]. The results obtained in these studies are described in more detail elsewhere [191,197,206,207].

Recently, in 2021, cemiplimab was introduced for first-line treatment for advanced NSCLC (EMPOWER-Lung 1) and, therefore, it is a subject of review herein [208]. Cemiplimab, a human IgG4 against PD1 mAb, was first approved in 2018 for the treatment of locally advanced and metastatic cutaneous squamous cell carcinoma (CSCC) patients who are not candidates for curative radiotherapy or surgery (NCT02383212 and NCT02760498) [209]. The approval was based on the results of two clinical trials involving 108 patients, which showed approximately half of the patients responded to the treatment. The treatment of cemiplimab was extended for use after first-line hedgehog inhibitor therapy (NCT03132636) [210]. Henceforth, cemiplimab can be used as a first-line treatment of advanced NSCLC patients with PD-L1 expression of a least 50%. A significant improvement was found in the overall survival and the progression-free survival (PFS) in comparison to chemotherapy. Among the 563 patients with PD-L1 of a least 50%, the median OS has not been yet reached (95% CI 17.9—not evaluable) with cemiplimab versus 14.2 months (95% CI, 11.2–17.5) with chemotherapy (hazard ratio (HR), 0.57; 95% CI, 0.42–0.77; *p* = 0.0002). The OS rates at 24 months were 50% and 27% in the investigate and the control arms, respectively. Moreover, the median PFS with cemiplimab was 8.2 months (95% CI, 6.1–8.8) versus 5.7 months (95% CI, 4.5–6.2) with chemotherapy, and the estimated PFS rates at 12 months were 21% and 7% in the investigative and the control arms, respectively. Patients treated with cemiplimab who had PD-L1 expression lower than 50% responded similarly to those treated with chemotherapy. The PD-L1 levels positively correlated with the improvements in OS and PFS. Adverse effects were observed in 28% and 39% of patients treated with cemiplimab and chemotherapy, respectively. Overall, cemiplimab was demonstrated to be a potential new treatment for NSCLC. ICIs antibodies can therefore inhibit PD-1/PD-L1 interaction or CTLA-4 immune checkpoints, improving antitumor immunity.

### 6.2. Combination Treatment Strategies

#### 6.2.1. Immune Checkpoint Inhibitor Combined with Chemotherapy

Chemotherapy treatment has shown that it can induce PD-L1 expression on tumor cells and, thus, the combination of immunotherapy and chemotherapy may produce a synergized effect and confer better survival outcome [211]. Chemotherapy is the first choice for patients that lack targetable driver mutations. Pembrolizumab targeting PD-1 was combined with chemotherapy in several clinical trials (KEYNOTE-021, KEYNOTE-189, and KEYNOTE 407). In 2018, the combination of pembrolizumab with pemetrexed and carboplatin was approved as a first-line treatment for metastatic non-squamous NSCLC patients with no driver mutation, irrespective of PD-L1 expression based on the results shown by KEYNOTE-021 [95]. A subsequent phase III trial concluded that the addition of pembrolizumab to chemotherapy resulted in longer OS and PFS than chemotherapy alone (KEYNOTE-189) [212]. Later, an expanded approval was obtained for the combination of pembrolizumab with carboplatin and placitaxel/nab-placitaxel for metastatic squamous NSCLC, irrespective of PD-L1 expression (Keynote-407) [213].

Several IMpower clinical trials demonstrated that atezolizumab combined with chemotherapy also produced synergistic effects and improved the efficacy over standard chemotherapy. Its combination with chemotherapy-based drugs (carboplatin, paclitaxel and bevacizumab) was approved by the FDA in 2018 for metastatic non-squamous NSCLC based on the IMpower150 phase III trial [214]. The quadrupole treatment prolonged PFS and OS for patients lacking *EGFR*/*ALK* mutations, independently of PD-L1 expression. The first phase III trial (IMpower130) demonstrated that atezolizumab combined with chemotherapy better improved the PFS and the OS compared to chemotherapy alone [215]. A second phase III trial showed no OS improvement when using combined treatment (atezolizumab plus carboplatin/nab-paclitaxel) for advanced-stage non-squamous NSCLC patients with no driver mutations but prolonged PFS (IMpower131) [216]. The next trial, the IMpower132 [217], used a combination of atezolizumab with pemetrexed and carboplatin/cisplatin and obtained similar conclusions as the IMpower131 trial. Moreover, atezolizumab combined with carboplatin and etoposide showed improved PFS and OS for first-line treatment of extensive-stage small cell lung cancer (IMpower133) [218]. As commented above, in 2021, cemiplimab was approved by the FDA for the treatment of patients with advanced NSCLC with PD-L1 expression of at least 50%. Moreover, a phase III trial (EMPOWER-Lung 3) showed a significantly improved OS in advanced or metastatic NSCLC by using first-line cemiplimab in combination with platinum chemotherapy (22 vs. 13 months, respectively). Additionally, this year, results from a phase III trial (POSEIDON) were released, which showed durvalumab, tremelimumab, and platinum-based chemotherapy provided OS benefit and significant improvement in PFS as compared to chemotherapy alone in metastatic NSCLC patients.

#### 6.2.2. Combined Immune Checkpoint Inhibitors: ICI PD-1/PD-L1 Combined with Anti-CTLA-4

In 2018, the efficacy of nivolumab in combination with ipilimumab, an ICI targeting CTLA4, was first demonstrated in a phase I trial (CheckMate 012) as a first-line treatment of advanced NSCLC [219]. A subsequent phase II trial (CheckMate 568) [220] identified tumor mutational burden (TMB) as a predictive biomarker to assess the efficacy of the combined therapy. Patients with TMB of 10 or more mutations/megabase were associated with improved response and prolonged PFS, independently of the PD-L1 expression. The phase III trial (CheckMate 227) observed a continued clinical benefit after 2 years of follow-up [221]. Durvalumab (ICI targeting PD-L1) with tremelimumab (a human monoclonal antibody against CTLA-4) was observed to improve the OS and the PFS in patients with metastatic NSCLC and PD-L1 expression lower than 25% (ARTIC trial) [222]. Another phase III trial (MYSTIC) considered patients with PD-L1 expression higher than 25% to evaluate the safety and the effectiveness of a dual immunotherapy combining durvalumab plus tremelimumab. In this study, there was no observed significant OS and PFS improvement with combined ICIs vs. chemotherapy [223]. However, patients with TMB of 20 or more mutations/megabase were identified with improved OS with the dual immunotherapy [224].

##### Combined Immune Checkpoint Inhibitors with EGFR-TKI

Durvalumab was also combined with osimertinib (a third generation EGFR-TKI) in a phase III trial (CAURAL), however, the clinical trial was terminated early because of increased incidence of interstitial lung disease-like events [104,225]. In the CheckMate 012 trial, nivolumab was combined with erlotinib in patients with EGFR-mutated advanced NSCLC [226]. Further studies are required to find appropriate target patients that may benefit from such combinations [227].

### 6.3. Predictive Biomarkers

Immunotherapy demonstrates great potential in canter treatment, and ICIs exhibit remarkable efficacy administered as monotherapy. Despite these achievements, only a small proportion of patients (~30%) benefit, and some of them develop resistance to anti-PD-1/PD-L1 immunotherapy [206]. Therefore, it is of great interest to identify biomarkers that can distinguish potential candidates that may benefit any immunotherapy or can predict effective responses to ICIs. The PD-L1 expression, the tumor mutational burden, and the MSI and/or the DNA MMR deficiency have been used as predictive biomarkers [180].

PD-L1 expression was shown as a predictive biomarker to select patients that can benefit from pembrolizumab treatment [228]. Immunohistochemistry (IHC) is currently used as a companion diagnostic test to estimate the expression levels of PD-L1, and several commercial kits for different epitopes were released (i.e., 22C3, 28-8, SP142, SP263, and 73-10) [229]. Each ICI uses a different antibody to estimate PD-L1 expression levels. Pembrolizumab uses 22C3 clone antibody, atezolizumab uses SP142 clone antibody, nivolumab uses 28-8 clone antibody, and durvalumab uses SP263 clone antibody. However, PD-L1 cannot yet be considered a fully sensitive and specific biomarker in clinical practice [207]. The lack of standardization of PD-L1 IHC assays represents a major source of uncertainty for PD-L1 testing. Moreover, the temporal and the spatial heterogeneity in the PD-L1 expression levels challenge its efficacy as a predictive biomarker. To date, only the 22C3 assay is required before initiating a first-line treatment with pembrolizumab monotherapy [230].

The measurement of MSI status and/or MMR deficiency was used as a predictive marker for response to PD-1 blockade by pembrolizumab [231]. Several clinical trials demonstrated a correlation between MMR deficiency and pembrolizumab efficacy for patients with multiple tumor types [232]. Consequently, pembrolizumab was approved by the FDA for the treatment of solid tumors with MMR deficiency [233]. Similar to PD-L1 expression, ICH is used for MMR detection. In particular, MSI needs to detect the expression of four MMR proteins (MLH1, MSH2, MSH6, and PMS2) [234]. MSI can be measured by PCR or NGS, the latter offering greater advantages over PCR-MS methods such as greater sensitivity or specificity.

TMB as MSI or MMR is an indicator of the genomic stability and is defined as the total number of mutations per megabase of DNA based on DNA sequencing [232,235]. New generation sequencing (NGS) technologies, including whole exome sequencing (WES) or large NGS panels, are used to determine the TMB. Based on the results in CheckMate 227 and CheckMate 026, TMB was suggested as a predictive biomarker for immunotherapy with nivolumab alone or in combination with ipilimumab [221]. However, TMB presents several limitations, including long test cycles, high cost, and standardization of the threshold for high- and low-TMB [230]. In 2020, TMB was approved by the FDA as a companion diagnostic biomarker for pembrolizumab [236].

## 7. Summary

We reviewed the latest research in global epidemiology, classification, molecular basis, targeted therapies, and immunotherapy in NSCLC. As the declining exposure of population to tobacco correlates with the economic development of particular countries, cigarette smoking seems to be a smaller and smaller issue to consider. Nevertheless, the exceeding levels of air particulates contamination are alarming due to their reported link to the growing incidence of respiratory track cancers. The high mortality observed in NSCLC patients indicates the need for early diagnosis. The implementation of molecular techniques allows us to understand the biology and the evolution of lung cancer as well as to find reliable biomarkers, improving its diagnosis. The application of tumor cell genotyping from the blood of patients contributes to the discovery of new or the assessment of the nature of already known key mutations of NSCLC. Unfortunately, the heterogeneous nature of lung tumors adds a level of complexity to its analysis. Deciphering cell molecular pathways and the recent technological development contributed to significant advancements in characterization, and organization, and tumor heterogeneity. The trend of personalized medicine has become a permanent feature in which the correlation between the histopathological diagnosis and the identification of driver mutations is an imperative for the individual choice of therapy for patients with NSCLC. Such treatment largely depends on the stage of the disease. However, low 5-year survival rates, even in patients treated at an early stage, are commonly found. Thus, the patients with an identified tumor molecular profile are advised to enroll in numerous clinical trials. Nowadays, a battery of drugs for targeted therapy is available for most of the mutated proteins (EGFR family, ALK, ROS1, NTRK, and RAF). Notwithstanding, the treatment for the K-Ras protein remains challenging. Fortunately, only in the past year, three inhibitors (pralsetinib, selpercatinib, and capmatinib) for targeted therapy and two antibody-based drugs (atezolizumab and combination of nivolumab and ipilimumab) were approved, giving hope for the development of effective treatment strategies for mutations such as the ones in the K-Ras protein. Immunotherapy emerged as an unexpected new weapon against NSCLC, and a new area of research was established. In 2015, nivolumab (anti PD-1 monoclonal antibody) was approved by the FDA as a second-line therapy for patients with advanced NSCLC. Then, other immune checkpoint inhibitors (ICIs) were successively introduced as first- and second-line monotherapy treatments or were combined with standard chemotherapy. Despite the clinical benefits of immunotherapy, a major challenge remains in the identification of patients that respond to ICIs or those that eventually do not respond anymore. A comprehensive summary of the current immunotherapies and the predictive biomarkers approved by the FDA and ongoing clinical trials was discussed above. To conclude, the landscape of therapies in NSCLC is rapidly evolving, and, thus, accurate and updated reviews are of utmost need.

## Figures and Tables

**Table 1:** Comparison of previous and new histopathological classifications of lung cancer, published by the World Health Organization [42]. While NSCLC does not exist in histopathological classification, in general use, it comprises: adenocarcinoma, squamous cell carcinoma, and large cell carcinoma.

Classification 2004		Classification 2015	
**Adenocarcinoma**	Adenocarcinoma, mixed subtype Acinar adenocarcinoma	**Adenocarcinoma**	Lepidic adenocarcinoma Acinar adenocarcinoma
	Papillary adenocarcinoma		Papillary adenocarcinoma
	Bronchioloalveolar carcinoma		Micropapillary adenocarcinoma
	Solid adenocarcinoma with mucin production		Solid adenocarcinoma
	Fetal adenocarcinoma		Invasive mucinous adenocarcinoma
	Mucinous cystadenocarcinoma		Colloid adenocarcinoma
	Mucinous (“colloid”) carcinoma		Fetal adenocarcinoma
	Signet ring adenocarcinoma		Enteric adenocarcinoma
	Clear cell adenocarcinoma		Minimally invasive adenocarcinoma
			Preinvasive lesions: adenocarcinoma in situ
**Squamous cell carcinoma**	Papillary	**Squamous cell carcinoma**	Keratinizing
	Clear cell		Nonkeratinizing
	Small cell		Basaloid
	Basaloid		Preinvasive lesions: Squamous cell carcinoma in situ
**Small cell carcinoma**	Combined small cell carcinoma	**Neuroendocrine tumors**	Small cell carcinoma
**Large cell carcinoma**	Large cell neuroendocrine carcinoma		Large cell neuroendocrine carcinoma
	Combined large cell neuroendocrine carcinoma		Carcinoid tumors
	Basaloid carcinoma		Preinvasive lesion
	Lymphoepithelioma-like carcinoma	**Large cell carcinoma**	
	Clear cell carcinoma	**Adenosquamous** **carcinoma**	
	Large cell carcinoma with rhabdoid phenotype	**Sarcomatoid carcinomas**	
**Adenosquamous carcinoma**		**Other and Unclassified carcinomas**	
**Sarcomatoid carcinoma**		**Salivary gland-type tumors**	
**Carcinoid tumor**		**Papillomas**	
**Salivary gland tumors**		**Adenomas**	

**Table 2:** FDA-approved targeted therapy drugs for the treatment of NSCLC.

Target	Inhibitor	Line of Treatment	Indication	Current-FDA Approval Year	Clinical Trial-Based Approval
EGFR	Gefitinib	first-line	metastatic NSCLC with exon 19 deletions or exon 21 (L858R) substitution mutations	2015	IFUM (NCT01203917)
	Erlotinib	first- or second-line	metastatic NSCLC with exon 19 deletions or exon 21 (L858R) substitution mutations	2016	IUNO trial (NCT01328951)
	Afatinib	first- or second-line treatment	metastatic NSCLC with non-resistant EGFR mutations; metastatic, squamous NSCLC progressing after platinum-based chemotherapy	2018	LUX-Lung 2 (NCT00525148), LUX-Lung 3 (NCT00949650), and LUX-Lung 6 (NCT01121393)
	Osimertinib	first-line or second- treatment	metastatic NSCLC with detected exon 19 deletions or exon 21 L858R mutations or T790M mutation-positive with disease progression on EGFR TKI therapy	2018	FLAURA, (NCT02296125)
	Dacomitinib	first-line	metastatic NSCLC with detected exon 19 deletions or exon 21 (L858R) substitution mutations	2018	ARCHER 1050 (NCT01774721)
ALK	Crizotinib	first-line	locally advanced or metastatic NSCLC	2011	PROFILE 1005 (NCT00932451)
	Ceritinib	first- or second-line	metastatic NSCLC	2017	ASCEND-4 (NCT01828099)
	Alectinib	first-line		2017	ALEX (NCT02075840)
	Brigatinib	second-line		2017	ALTA (NCT02094573)
	Lorlatinib	second- or third line	metastatic NSCLC after progression on other ALK TKI therapy	2018	Study B7461001 (NCT01970865)
ROS1	Crizotinib	first-line	metastatic NSCLC	2016	PROFILE 1001 (NCT00585195)
	Entrectinib	first-line		2019	STARTRK-1 (NCT02097810) STARTRK-2 (NCT02568267)
NTRK	Larotrectinib	first-line	solid tumors with detected *NTRK* gene fusion without a known acquired resistance mutation, independent of tumor origin	2018	LOXO-TRK-14001 (NCT02122913), SCOUT (NCT02637687), NAVIGATE (NCT02576431)
	Entrectinib	first-line		2019	STARTRK-1 (NCT02097810) STARTRK-2 (NCT02568267)
RET	Pralsetinib	first-line	metastatic NSCLC	2020	ARROW (NCT03037385)
	Selpercatinib	first-line	metastatic NSCLC	2020	LIBRETTO-001 (NCT03157128)
MET	Capmatinib	first-line	metastatic NSCLC with specific mutations (exon 14 skipping)	2020	GEOMETRY (NCT02414139)
	Tepotinib	first-line		2021	VISION (NCT02864992)

**Table 3:** FDA-approved immunotherapy drugs for the treatment of NSCLC.

Checkpoint Inhibitor	Target	Line of Treatment	Indications	Clinical Trial-Based Approval	FDA Approval Year
Nivolumab	PD-1	second-line	metastatic squamous NSCLC after chemotherapy;	CheckMate 017 (NCT01642004)	2015
		second-line	extension to non-squamous NSCLC;	CheckMate 057 (NCT01673867)	
Pembrolizumab	PD-1	first-line	metastatic NSCLC; with no EGFR or ALK mutation; TPS ≥ 50%;	KEYNOTE−024 (NCT02142738)	2016
		second-line	progression after chemotherapy or TKI in metastatic NSCLC; with TPS ≥ 1%;	KEYNOTE-010 (NCT01905657)	
		first-line	unresectable stage III or metastatic NSCLC; no possible definitive chemoradiation; with no EGFR or ALK mutation; TPS ≥ 1%;	KEYNOTE-042 (NCT02220894)	2019
Atezolizumab	PDL-1	second-line	metastatic NSCLC with progression on/after chemotherapy or TKIs;	OAK (NCT02008227) POLAR (NCT01903993)	2016
		first-line	combined with chemotherapy; metastatic non-squamous NSCLC; with no EGFR or ALK mutation;	IMpower150 (NCT02366143)	2018
Durvalumab	PDL-1	second-line	unresectable Stage III NSCLC; with no progression after chemoradiation therapy;	PACIFIC (NCT02125461)	2018
Ipilimumab	CTLA-4	first-line	only in the combination with nivolumab; metastatic NSCLC; with no EGFR or ALK mutation; TPS ≥ 1%;	CheckMate 227 (NCT02477826)	2020
Cemiplimab	PD-1	first-line	advanced NSCLC; TPS ≥ 50%	EMPOWER-Lung 1 (NCT03088540)	2021
